# Electromyography data for non-invasive naturally-controlled robotic hand prostheses

**DOI:** 10.1038/sdata.2014.53

**Published:** 2014-12-23

**Authors:** Manfredo Atzori, Arjan Gijsberts, Claudio Castellini, Barbara Caputo, Anne-Gabrielle Mittaz Hager, Simone Elsig, Giorgio Giatsidis, Franco Bassetto, Henning Müller

**Affiliations:** 1 Information Systems Institute at the University of Applied Sciences Western Switzerland (HES-SO Valais), Technoark 3, 3960 Sierre, Switzerland; 2 Institute de Recherche Idiap, Rue Marconi 19, 1920 Martigny, Switzerland; 3 Robotics and Mechatronics Center, DLR—German Aerospace Center, Muenchener Strasse 20, 82234 Oberpfaffenhofen, Germany; 4 Department of Computer, Control, and Management Engineering, University of Rome La Sapienza, via Ariosto 25, 00185 Roma, Italy; 5 Department of Physical Therapy at the University of Applied Sciences Western Switzerland (HES-SO Valais), Rathausstrasse 8, 3954 Leukerbad, Switzerland; 6 Clinic of Plastic Surgery, Padova University Hospital, Via Giustiniani 2, 35128 Padova, Italy

## Abstract

Recent advances in rehabilitation robotics suggest that it may be possible for hand-amputated subjects to recover at least a significant part of the lost hand functionality. The control of robotic prosthetic hands using non-invasive techniques is still a challenge in real life: myoelectric prostheses give limited control capabilities, the control is often unnatural and must be learned through long training times. Meanwhile, scientific literature results are promising but they are still far from fulfilling real-life needs. This work aims to close this gap by allowing worldwide research groups to develop and test movement recognition and force control algorithms on a benchmark scientific database. The database is targeted at studying the relationship between surface electromyography, hand kinematics and hand forces, with the final goal of developing non-invasive, naturally controlled, robotic hand prostheses. The validation section verifies that the data are similar to data acquired in real-life conditions, and that recognition of different hand tasks by applying state-of-the-art signal features and machine-learning algorithms is possible.

## Background & Summary

The recent, rapid evolution of portable sensors and mechatronic technology has made robotics available in everyday life. The application of these advancements of the field of prosthetics could greatly impact the quality of life of impaired people, but still faces many challenges. The work described in this paper aims to aid the progress in robotic hand prosthetics, making it possible to develop and test algorithms for movement recognition and force control on a scientific benchmark database.

Currently, trans-radial amputated subjects (who are the majority of upper limb amputated people^1^) can rely on myoelectric (controlled by electromyography, sEMG) prostheses. In most cases the tasks that a prosthesis can perform are limited to opening and closing, but in recent years top-level commercial offers have appeared, including mechanically advanced prostheses that can perform several programmable movements. However, the methods used to control such advanced hands are usually rudimentary, relying on sequential control strategies. Controlling the prosthesis is far from natural motion, and the user must undergo a long and complicated training procedure. Myoelectric prostheses could potentially improve the quality of life of hand amputees, but the control system hinders this advancement and it is one of the main causes of the low acceptance of such devices^1^.

Improvements over the conventional myoelectric control strategy have already been described in scientific literature^2–11^. Excellent results have been obtained with invasive methods^9^, while non-invasive studies usually show average classification accuracies of hand movements up to 80–90% (ref. 8), exceeding 90% in specific cases^2,7^. Often, non-invasive methods are based on the use of several electrodes to record sEMG, and pattern recognition algorithms to classify the movement that the subject is willing to perform, and recently, such a system has been clinically deployed (www.coaptengineering.com). Nevertheless, research in this field still suffers from a number of problems. First, the described studies usually include too few subjects (according to our knowledge, up to 11 intact subjects and 6 amputees^12^) and too few tasks (according to our knowledge, up to 12 (ref. 5)), which makes it difficult to obtain statistically relevant results. Second, it is so far unknown how clinical parameters related to the amputation (for example, remaining forearm percentage, phantom limb sensation, use of prostheses^7,13^) and physiological phenomena (such as cortical reorganization) can affect the natural control capability of the prosthesis. Lastly, the movement recognition accuracy is never high enough to avoid misclassification on a large number of movements, which is paramount in real-life.

Moreover direct quantitative comparison among methods is usually difficult since publicly available data collections are extremely rare^14,15^. In contrast with this situation, the importance of solid benchmarking protocols and publicly available databases has been confirmed repeatedly in several fields^16,17^, where it contributed to promote comparison between methods and was an impetus for progress.

In this work we describe the *Ninapro* (Non Invasive Adaptive Prosthetics) database (Data Citations 1 and 2), which includes data acquired from 67 intact subjects and 11 hand-amputated subjects while performing several repetitive tasks such as hand movements and finger force patterns. The database aims at allowing worldwide research groups to study the relationship between sEMG, hand/arm kinematics and dynamics, and clinical parameters, with the final goal of creating non-invasive, naturally controlled robotic hand prostheses for trans-radial amputees. The number of subjects who participated in the data collection is comparatively high, especially considering the difficulty of recruiting trans-radial amputees, and the fact that intact subjects can only be used as a ‘proxy’ measure for amputees^18^. Some parts of the database have already been used in traditional scientific papers on intact subjects^14,19^ with the aim of characterizing pre-processing and classification procedures, clinical parameters, and introducing the *Movement Error Rate* as an alternative to the standard window-based accuracy.

This data-description paper introduces the full database, advancing the state of the art thanks to a comparative and unique description of all the data, and it applies new analysis methods, thus being the most accurate, comprehensive and advanced reference for the largest sEMG database existing at the time of writing to the best of our knowledge. Moreover, the technical validation section verifies that the data are similar to data acquired in real-life conditions and that they permit recognition of different hand movements by applying state-of-the-art signal features and machine-learning algorithms.

## Methods

### Subjects and ethical requirements

We tested 78 subjects (67 intact subjects, 11 trans-radial amputated subjects) whose data are split across three sub-databases, according to the acquisition procedure and subject characteristics (Data Citation 1,Table 1). The first database contains data obtained from 27 intact subjects (20 males, 7 females; 25 right handed, 2 left handed; age 28±3.4 years). The second database contains data obtained from 40 intact subjects (28 males, 12 females; 34 right handed, 6 left handed; age 29.9±3.9 years). The third database contains data obtained from 11 trans-radial amputated subjects (11 males; 10 right handed, 1 left handed; age 42.36±11.96 years). (More details about the subjects are reported in Table 2.)

Before the data acquisition began, each subject was given a thorough written and oral explanation of the experiment itself, including the associated risks; the subject would then sign an informed consent form. The experiment was conducted according to the principles expressed in the Declaration of Helsinki (www.wma.net/en/20activities/10ethics/10helsinki) and it was approved by the Ethics Commission of the Canton Valais (Switzerland).

### Acquisition setup

The acquisition setup included several sensors, designed to record hand kinematics, dynamics and the corresponding muscular activity. The sensors were connected to a laptop responsible for data acquisition.

Hand kinematics was measured using a 22-sensor *CyberGlove II* dataglove (CyberGlove Systems LLC, www.cyberglovesystems.com). The CyberGlove is a motion capture data glove, instrumented with joint-angle measurements. It uses proprietary resistive bend-sensing technology to accurately transform hand and finger motions into real-time digital joint-angle data. The device returns 22 8-bit values proportional to these angles for an average resolution of less than one degree depending on the size of the subject’s hand. In addition to the CyberGlove, a standard commercially available 2-axis *IS40* inclinometer (Fritz Kübler GmbH, www.kuebler.com) was fixed to the subject’s wrist to measure the wrist orientation. This inclinometer has a range of 120° and a resolution of less than 0.15°.

Hand dynamics was measured using the *Finger-Force Linear Sensor* (FFLS)^20^, employing strain gauge sensors to detect flexion and extension forces of all fingers, plus abduction and adduction of the thumb. This sensor is characterized by high signal repeatability, minimal drift over time, almost perfect linearity and virtually no hysteresis (both parameters have a maximum deviation of 0.3%).

Muscular activity was measured using either OttoBock or Delsys double-differential sEMG electrodes. In the first configuration ten *MyoBock 13E200-50* electrodes (Otto Bock HealthCare GmbH, www.ottobock.com) were used, providing an amplified, bandpass-filtered and Root-Mean-Square (RMS) rectified version of the raw sEMG signal. The electrodes’ amplification gain was set at about 14,000. These electrodes are already widely used in prosthetics; they employ frequency shielding and filtering in order to avoid low and high frequency interferences emitted, for example by 50–60 Hz power sources, mobile phones or security systems. These electrodes were fixed on the forearm using an elastic armband. In the second configuration we used 12 *Trigno Wireless* electrodes (Delsys, Inc, www.delsys.com), each one equipped with a self-contained rechargeable battery and with an operational range of 40 m (the setup also includes a wireless receiving base station). sEMG signals are sampled at a rate of 2 kHz with a baseline noise of less than 750 nV RMS. These electrodes also integrate a 3-axes accelerometer sampled at 148 Hz and were fixed on the forearm using their standard adhesive bands. A hypoallergenic elastic latex–free band was placed around the electrodes to keep them fixed during the acquisition.

**Figure 1:**
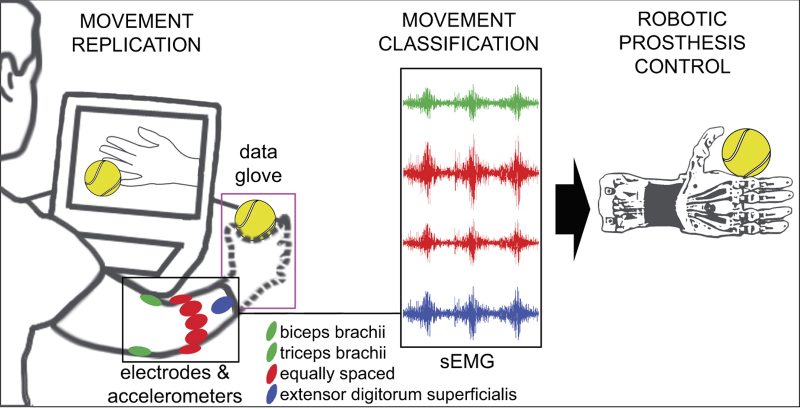
Acquisition procedure scheme for exercises A, B and C. The subjects are asked to mimic movies of movement shown on the screen of the laptop. The sEMG signal is recorded through up to 12 electrodes and can be used to test methods to control robotic hand prostheses naturally (the electrode on the flexor digitorum superficialis is not represented due to perspective reasons).

Particular care was taken in the placement of the electrodes on the forearm, since this is usually regarded as a crucial step for data usability. We decided to combine two methods which are common in the field, that is, a dense sampling approach^5,6,21^ and a precise anatomical positioning strategy^22,23^. The electrodes are positioned as shown in Fig. 1: eight electrodes are equally spaced around the forearm at the height of the radio-humeral joint; two electrodes are placed on the main activity spots of the flexor digitorum superficialis and of the extensor digitorum superficialis^14^; in the second configuration only, two electrodes were also placed on the main activity spots of the biceps brachii and of the triceps brachii. The main activity spots were identified by palpation. This positioning of the electrodes also gives the opportunity to improve inter-subject classification results by applying linear and non-linear spatial registration algorithms, as described in Atzori *et al.*^24^

Data from all sensors were transmitted to the laptop used for data acquisition in different ways. Data from the CyberGlove were transmitted over a Bluetooth-tunneled serial port at slightly less than 25 Hz; data from the inclinometer, the FFLS and the Otto-Bock sEMG electrodes were acquired at 100 Hz using a National Instruments data acquisition card (NI-DAQ PCMCIA 6024E, 12-bit resolution); the Delsys base station received the sEMG and accelerometer streams via an *ad-hoc* wireless network and relayed the data via a standard USB connection to the laptop. Each data sample provided by each sensor was associated to an accurate timestamp using Windows performance counters.

### Acquisition protocol

Preceding the experiment, each subject is requested to give informed consent and to answer questions including age, gender, height, weight and laterality. In the case of amputees, we also note the date, type and reason of the amputation, remaining forearm percentage, information about the use of prostheses (cosmetic, kinematic, myoelectric), type and degree of phantom limb sensation and DASH (Disability of the Arm, Shoulder and Hand) score^25^. The remaining forearm percentage is computed as the ratio between the length of the amputated forearm and the length of the contralateral forearm from the elbow to the wrist, rounded to the tens. Subsequently, subjects were made to sit at a desk on an office chair, adjusted to match the maximum comfort, and comfortably resting their arms on the desktop. A laptop in front of the subject provided visual stimuli for each task, while also recording data from the measurement devices.

**Figure 2:**
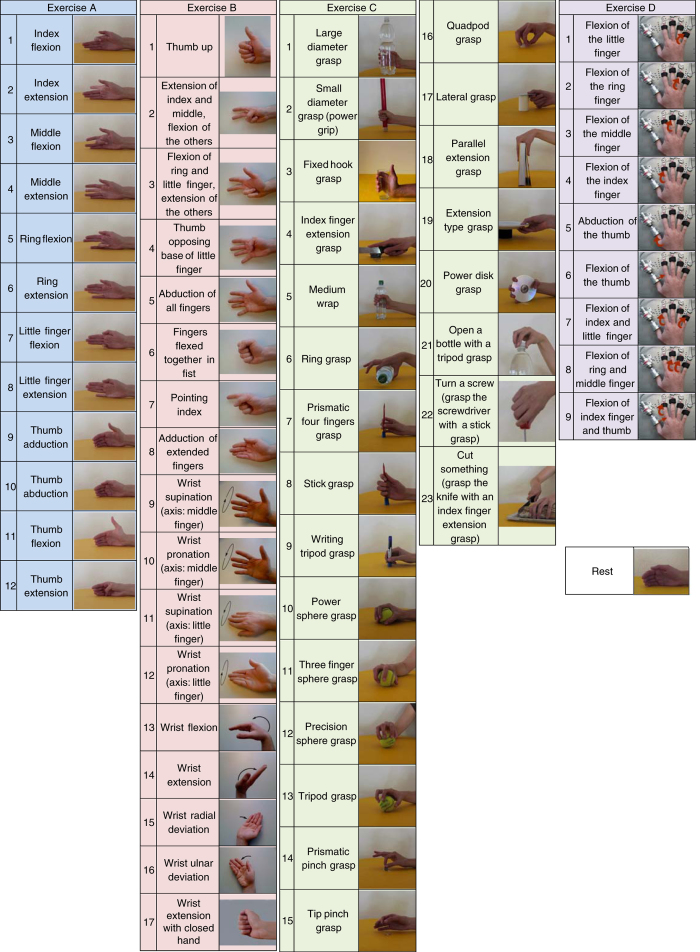
Movements and force patterns divided by exercise. Exercise A (light blue): 12 basic movements of the fingers; Exercise B (red): 8 isometric and isotonic hand configurations and 9 basic movements of the wrist; Exercise C (green): 23 grasping and functional movements (everyday objects are presented to the subject for grasping, in order to mimic daily-life actions); Exercise D (purple): 9 force patterns; Rest position (white).

The experiment is divided into one training part and three exercises addressing different types of movements (Fig. 2), interrupted by rest time in order to avoid muscular fatigue. The training phase consisted of a condensed mix of the exercises, in order for the subjects to become familiar with the experiment.

The details of the acquisition procedure depend on the kinematics or dynamics acquisition setup. During the exercises performed using the Cyberglove II, the intact subjects were asked to mimic movies of movement shown on the screen of the laptop with their right hand, while amputated subjects were asked to mimic the movements with the missing limb as naturally as possible (Fig. 1). Since the main aim of kinematics data is to permit movement classification, all the subjects were asked to concentrate on mimicking the movements rather than on exerting high forces. The set of movements was selected from the hand taxonomy, robotics, and rehabilitation literature^4,12,26–30^ with the aim of covering the majority of the hand movements encountered in activities of daily living (ADL). Each movement repetition lasted 5 s, and it was alternated with a rest posture lasting 3 s. The sequence of movements was not randomized in order to encourage repetitive, almost unconscious movements.

During the exercises performed using the FFLS, subjects were instructed to repeat nine force patterns (Fig. 2) by pressing with one or more right hand digits on the device. An initial calibration phase was performed to establish the rest and maximal voluntary contraction (MVC) force levels for all fingers, and training was performed before each force pattern. (The MVC for the amputated limb was estimated according to the sensation of the subject.) The force levels requested for each finger were represented as coloured bars on the screen. During the exercise, the stimulus increased up to 80% of the maximal voluntary contraction force established during calibration, and then it decreased to 0% following a squared-sinusoidal pattern.

Also in this case, intact subjects were asked to execute the experiment with their right hand, while amputated subjects were asked to think to repeat the movements as naturally as possible with the missing limb. It is important to remark that amputees cannot, in general, produce any reliable ground truth due to the inability to operate any sensor with the missing limb. In related literature, this fundamentally unsolvable problem has been circumvented either by (a) instructing the subjects to execute a task bilaterally while recording the ground truth from the intact limb^31^; or by (b) asking them to follow a visual stimulus (either on a screen^31,32^ or performed by the experimenter^31^).

There is no consensus on the best procedure, so each subject was left free to choose after a short training phase, which resulted in only two subjects undergoing bilateral execution. As a result of this, for the rest of the amputees, the database contains only the stimulus as ground truth. Analyses with the stimulus as ground truth have, anyway, already been successfully performed (for example, see refs. 31–33).

### Signal processing

Several signal processing steps were performed before making data publicly available on the repositories (Data Citations 1 and 2). These steps included synchronization, relabelling and (for the Delsys electrodes) filtering. The raw data are available upon request.

#### Synchronization

high-resolution timestamps were used to synchronize the data streams. Specifically, all streams were super-sampled to the highest sampling frequency (2 kHz or 100 Hz, depending on the used sEMG electrodes) using linear interpolation (real-valued streams) or nearest-neighbour interpolation (discrete streams).

#### Relabelling

The movements performed by the subjects may not perfectly match with the stimuli proposed by our software due to human reaction times and experimental conditions. The resulting erroneous movement labels have been corrected by applying a generalized likelihood ratio algorithm^34^ offline, which realigns the movement boundaries by maximizing the likelihood of a rest-movement-rest sequence. Both the original labels and the new labels are included in the files.

#### Filtering

The Delsys electrodes are not shielded against power line interferences, which can affect the recoded signal in particular cases. Therefore, prior to synchronization, the Delsys sEMG signals were cleaned from 50 Hz (and harmonics) power-line interference using a Hampel filter^34^.

## Data Records

The data produced with the described methods have been stored in two online repositories. The first is the official Ninapro repository (Data Citation 1), which also gives the opportunity to upload classification results for each database, together with details regarding the classification procedure. The second is Dryad (Data Citation 2), which is a general-purpose resource that makes the data underlying scientific publications discoverable, freely reusable, and citable. The format and content for both data sets are described below.

For each subject and exercise, the database contains one file in *Matlab* format (www.mathworks.com) with synchronized variables. The variables included in the files are:

- subject: subject number;
- exercise: exercise number;
- emg: sEMG signal of the electrodes; columns 1–8 include the signal from the electrodes equally spaced around the forearm; columns 9 and 10 include the signal from the electrodes located on the main activity spots of the *muscle Flexor Digitorum Superficialis* and of the *muscle Extensor Digitorum Superficialis*^14^; when available, columns 11 and 12 include the signal from the main activity spots of the *muscle Biceps Brachii* and of the muscle *Triceps Brachii;*
- acc (36 columns): (x,y,z)-axis acceleration values of the 12 electrodes;
- glove (22 columns): uncalibrated signal from the 22 sensors of the Cyberglove. The raw data are declared to be proportional to the angles of the joints in the CyberGlove manual; details on the location of the sensors are available at the link: ninapro.hevs.ch/node/123;
- inclin (2 columns): inclinometer (roll,pitch) values;
- stimulus (1 column): the original label of the movement repeated by the subject;
- restimulus (1 column): the a-posteriori refined label of the movement;
- repetition (1 column): stimulus repetition index;
- rerepetition (1 column): restimulus repetition index;
- force (6 columns): force values;
- forcecal (2×6 values): maximal force values (minimal and maximal force values for each sensor).

The raw unsynchronized data are also available upon request.

## Technical Validation

The Ninapro data (Data Citations 1 and 2) should be statistically as similar as possible to data acquired in real life, especially from amputees, and they should make it possible to recognise different hand movements with results comparable with other works described in scientific literature.

To verify that the data are similar to data produced in real life, we evaluate the effect of experimental conditions on the amplitude of the signals.

To verify that the data allow the recognition of hand movements, we apply four state-of-the-art classification methods on five signal features using an approach that is very common in the field of sEMG. In this section, we also compare the classification accuracy obtained on subsets of movements that were previously described in literature^5,6^.

### Experimental condition effect on signal amplitude

**Figure 3:**
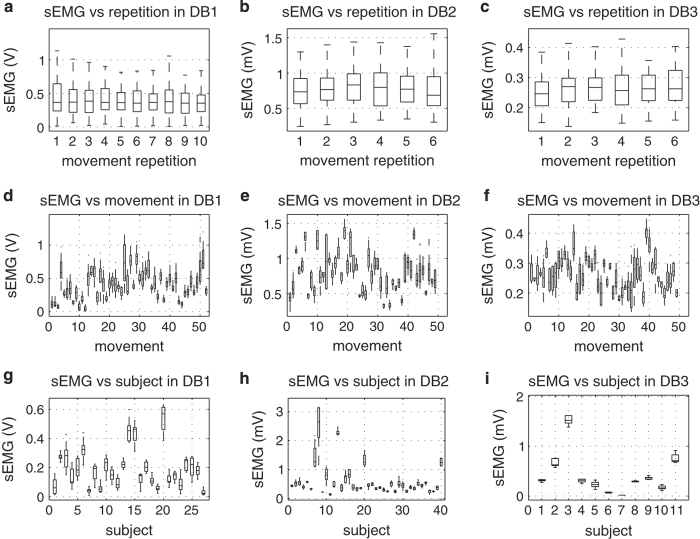
Experimental conditions effect on muscular activity for different sub-databases. Different rows represent different experimental conditions: movement repetition (1st row; subplots **a**–**c**); movement (2nd row; subplots **d**–**f**); subject (3rd row; subplots **g**–**i**). Different columns represent different sub-databases: database 1 (1st column; subplots **a**,**d**,**g**); database 2 (2nd column; subplots **b**,**e**,**h**); database 3 (3rd column; subplots **c**,**f**,**i**). The horizontal central mark in the boxes is the median; the edges of the boxes are the 25th and 75th percentiles; the whiskers extend to approximately 2.7 times the standard deviation.

**Figure 4:**
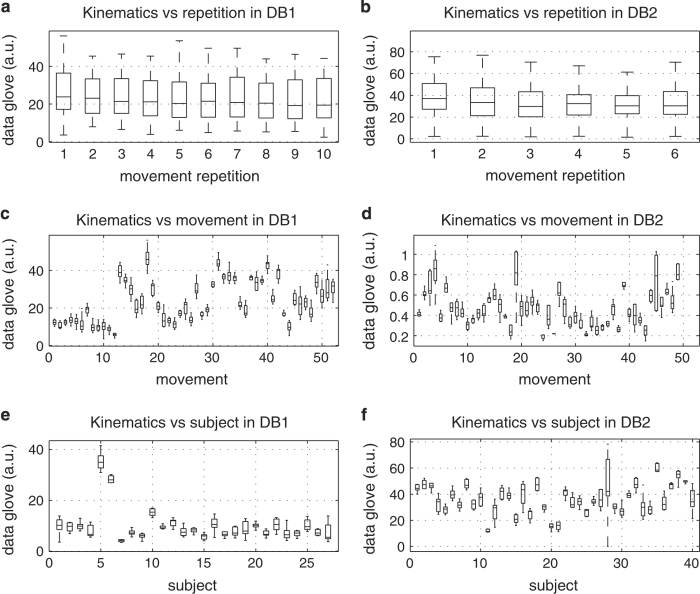
Experimental conditions effect on hand kinematics for different sub-databases. Different rows represent different experimental conditions: movement repetition (1st row; subplots **a**,**b**); movement (2nd row; subplots **c**,**d**); subject (3rd row; subplots **e**,**f**). Different columns represent different sub-databases: database 1 (1st column; subplots **a**,**c**,**e**); database 2 (2nd column; subplots **b**,**d**,**f**). The horizontal central mark in the boxes is the median; the edges of the boxes are the 25th and 75th percentiles; the whiskers extend to approximately 2.7 times the standard deviation.

**Figure 5:**
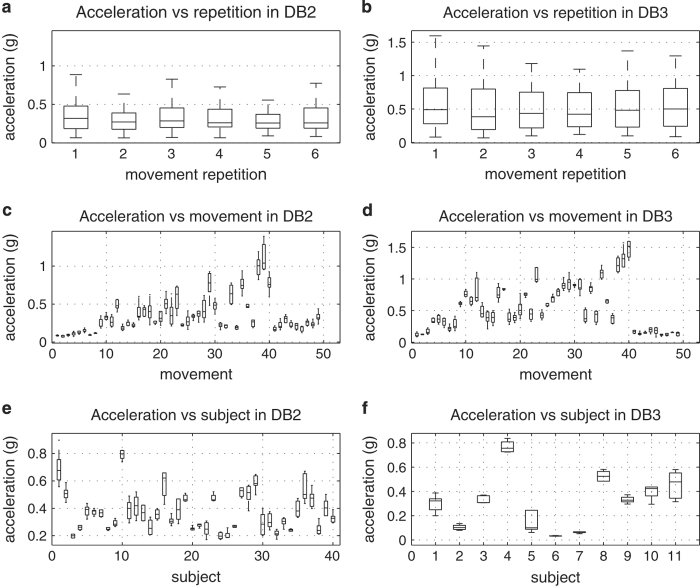
Experimental conditions effect on acceleration sensors for different sub-databases. Different rows represent different experimental conditions: movement repetition (1st row; subplots **a**,**b**); movement (2nd row; subplots **c**,**d**); subject (3rd row; subplots **e**,**f**). Different columns represent different sub-databases: database 2 (1st column; subplots **a**,**c**,**e**); database 3 (2nd column; subplots **b**,**d**,**f**). The horizontal central mark in the boxes is the median; the edges of the boxes are the 25th and 75th percentiles; the whiskers extend to approximately 2.7 times the standard deviation.

To ensure the quality of the Ninapro data (Data Citations 1 and 2), we evaluate the effect of experimental conditions (movement repetition, movement number and subject number) on the signal. In particular, we consider the amplitude of the sEMG signal (Fig. 3), of the data glove (as an estimate of hand kinematics) (Fig. 4) and of the acceleration (Fig. 5). The test was performed with a one-way Multivariate Analysis of Variance (MANOVA) using Matlab *(Mathworks)*.

Many factors can affect the amplitude of the signal from sEMG electrodes^35,36^ and the other sensors, including the acquisition setup, the anatomical characteristics of the subject, fatigue and (for amputated subjects) clinical parameters related to the amputation. In all cases but two, the results show that there are not significant differences considering the number of movement repetitions (*P*>0.1), while there are significant differences considering different subjects and movements. This is acceptable considering that different subjects are characterized by different anatomical features, different experience as prosthetic users and muscle/stump fitness, and considering that different movements involve the use of different muscles, different objects and different force patterns, thus leading to different average sEMG and kinematic signal magnitudes.

The only exceptions are given by the analysis of the relationship between hand kinematic signal and movement repetition (*P*<0.01) (Fig. 4a,b). These results, together with the visual inspection of the plots, suggest that in some cases there can be a statistically significant relationship between the signal amplitude and the movement repetition. We tested this by linear regression on the mean amplitude of sEMG, data glove and accelerometers analysing each subject separately. Considering the sEMG, a significant (*P*<0.05) dependence on the repetition is obtained in 25.93% (database 1), 12.5% (database 2) and 9% (database 3) of the subjects. Considering the data glove, a significant (*P*<0.05) dependence on the repetition is obtained in 66.7% (database 1) and 35% (database 2—no dependence found in database 3) of the subjects. Considering the accelerometers, a significant (*P*<0.05) dependence on the repetition is obtained in 15% of cases in database 2 (but it should be noted that accelerometers were not recorded in database 1). This result can be related to neuromuscular adaptation to the movement. Despite this, the effect seems to be significant only in a few subjects (especially considering sEMG); care should be taken when splitting movement repetitions for movement classification.

### Movement classification

#### (1) Feature extraction and classification procedure

The classification procedure follows Englehart *et al.*^19,37^ and consists of pre-processing, windowing (at 200 ms), feature extraction and classification. Approximately one third of the movement repetitions were used to create the test set (repetition 2, 5 and 7 in database 1; repetition 2 and 5 in database 2 and database 3, Data Citations 1 and 2), while the remaining repetitions were used to create the training set.

We considered five signal features and four classification methods, selected upon previous application to sEMG and popularity.

The selected signal features are: Root-Mean-Square (RMS), the time domain statistics described by Hudgins *et al.*^38^ (TD), Histogram (HIST), marginal Discrete Wavelet Transform (mDWT) and the normalized combination of all of the above. All the features have been applied successfully to myoelectric signals^19,34,37^. While using Histogram (HIST)^39^, the histogram was divided into 20 bins along a 3σ threshold. For the marginal Discrete Wavelet Transform (mDWT), we used a db7 wavelet with three levels^40^. The classifiers that we used are well known and have been applied in other fields of machine learning, including sEMG analysis. They include: k-Nearest Neighbors^41^, Support Vector Machines (SVM)^42^ and Random Forests^43^. The classification was performed on all movements (rest included) and is balanced according to movement number repetitions. Before performing the classification, the data from database 1 were preprocessed using a 1st order Butterworth low-pass filter with a 1 Hz cutoff frequency^44^.

#### (2) Classification of all movements

**Figure 6:**
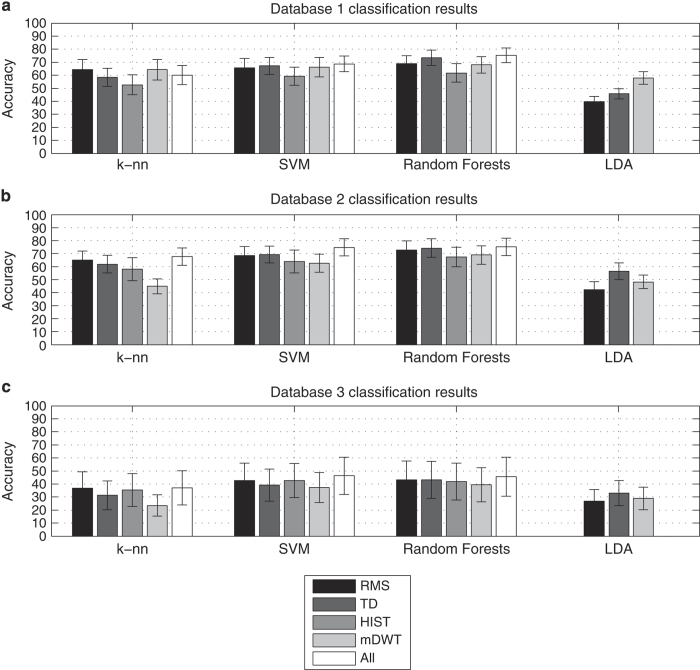
Movement classification results for different sub-databases, classifiers and features. Different histograms represent different databases: (**a**) database 1; (**b**) database 2; (**c**) database 3. Each group of columns represents a specific classifier (k-nn, k-nearest neighbors; SVM, Support Vector Machine; random forests; LDA, Linear Discriminant Analysis). Different colours represent different features (RMS, Root Mean Square; TD, time domain statistics; HIST, Histogram; mDWT, marginal Discrete Wavelet Transform, normalized combination of all features). The height of each column represents the average accuracy, while the error bar represents the standard deviation.

As can be seen in Fig. 6, the classification results from intact subjects are similar (database 1 and database 2), while they are approximately 20% higher than the results obtained from amputated subjects (database 3). In all the cases, the results are much higher than the chance level, which is 1.89% for database 1 and 2% for database 2 and database 3.

For DB1, the highest average classification accuracy for 50 movements is 75.32%, obtained with Random Forests and all features. For DB2, the highest average classification accuracy for 50 movements is 75.27%, obtained with Random Forests and all features.

For amputated subjects, the highest average classification accuracy for 50 movements is 46.27%, obtained with SVM and all features. The ratio between the accuracy and the chance level is higher in this case than in previous results described in the literature for similar tasks, for example, 8.5 (10 movements, accuracy 84.4% (ref. 6), 10.56 (12 movements, accuracy 87.8% (5)). This is probably due to the number of considered movements or to differences in the analysis procedure.

#### (2) Classification of subsets of movements

The first comparison on a subset of movements was performed on intact subjects using as reference the paper by Tenore *et al.*^5^, while the second was performed on amputated subjects using as reference the paper by Li *et al.*^6^ In both cases, it must be noted that the acquisition setup and the protocol are substantially different, so the classification accuracy results should be regarded as a qualitative evaluation of the database rather than a quantitative one. The Ninapro acquisition protocol includes at least 50 different movements, while the considered references include respectively 13 and 11 movements. Therefore, each movement in Ninapro could be repeated only up to 6 times in order to avoid fatigue in the subjects, while the number of movement repetitions in the reference papers are respectively 25–30 and 16. This difference is particularly important because it can strongly affect classification accuracy.

The comparison with Tenore *et al.*^5^ was performed on the intact subjects included in DB1. The considered movements are: 1, 2, 3, 4, 5, 6, 7, 8, 11, 12 from exercise A and movement 5 and 7 from exercise B. In order to make the classification procedure as similar as possible in the two cases, the time domain statistics feature was used in combination with a leave-one-out approach. Moreover, the SVM classifier was chosen, since it gave results similar to multilayer perceptrons (MLP) on DB1^34^. The average classification accuracy obtained is (82.77±9.27)%, which is similar to the average classification accuracy for Tenore (89.6±5)%. In this case, it should also be noted that we used commercial electrodes that record rectified (instead of raw) signal, since this factor can affect the classification result as well.

The comparison with Li *et al.*^6^ was performed on four of the amputated subjects included in DB3, selected in order to have amputation levels similar to those described in the reference paper (forearm percentage >70%). The considered movements are: 5, 11, 12, 13 and 14 from exercise B and movement 2, 4, 15, 16 and 17 from exercise C. Also in this case, in order to make the classification procedure as similar as possible in the two cases, the time domain statistics feature was used in combination with a leave-one-out approach. Moreover, the LDA classifier was chosen, as in the reference paper. The average classification accuracy obtained is (61.38±9.56)%, which is inferior to the average classification accuracy described by Li (79±11)%. Several factors can determine the classification accuracy difference. First of all, the subjects described by Li had been pre-trained before, and the accuracy from the first acquisition was approximately 5% inferior^6^. Second, the data in the reference paper have 28% more training sets. Third, Li *et al.* had more electrodes placed directly on the forearm. Fourth, the movements are not entirely equal. Last but definitely not least, the subjects were different, and we strongly believe that several clinical parameters can affect the capability of hand amputated subjects to reproduce movements.

Despite some differences from previous literature, it is evident from these results that the data can be used for movement recognition analysis in hand-amputated subjects to improve the state of the art in robotic hand prostheses and can also offer a baseline for future studies with the Ninapro repository (Data Citations 1 and 2).

## Usage Notes

There are several potential uses for this database. The main one is the application of pattern recognition and machine learning methods to recognize movements in the sEMG signal in order to compare different signal features and methods. We encourage any use that can contribute to the creation of non-invasive, naturally controlled robotic hand prostheses for trans-radial amputees, but the database could also be used to study hand kinematics and dynamics in intact subjects (for example, in order to control exoskeletons or robotic hands), as well as for other aims.

In any use involving the amputated subjects (database 3), the users should keep in mind that in two amputated subjects (subjects 7 and 8) the number of electrodes was reduced to ten due to insufficient space and that three amputated subjects (subjects 1, 3 and 10) asked to interrupt the experiment before its end due to fatigue or pain. These subjects performed respectively 39, 49 and 43 movements (including rest), and are included in database 3.

In any use involving hand kinematics data, the users should keep in mind that the Cyberglove II data represent the raw sensor values rather than estimated joint angles, the reason being that reliable calibration of the glove is prohibitively time consuming. Usually this should not be a problem, since most machine learning techniques are invariant to linear scaling of the data and calibration is in these cases unnecessary; moreover this decision gives more data processing freedom to the final users. If desired, exact joint angles can be obtained by calibrating the glove a posteriori for a given subject.

The technical validation described in this paper suggests that in some cases there can be a relationship between the signal amplitude and movement repetition. This effect seems to be significant only in few subjects (especially considering sEMG), however attention should be paid to it while splitting movement repetitions into training set and test set for movement classification.

Often, the classification results described in the literature are unbalanced, and even more often it is not mentioned if the results are balanced or not. When using the Ninapro database this point should always be described in detail in order to permit comparisons between different studies and methodologies (as previously described by Atzori *et al.*^14^).

## Additional information

**How to cite this article:** Atzori, M. *et al.* Electromyography data for non-invasive naturally-controlled robotic hand prostheses. *Sci. Data* 1:140053 doi: 10.1038/sdata.2014.53 (2014).

## Supplementary Material



## Figures and Tables

**Table 1:** Ninapro database summary table.

	**Database 1**	**Database 2**	**Database 3**
Intact Subjects	27	40	0
Trans-radial Amputated Subjects	0	0	11
sEMG Electrodes	10 Otto Bock	12 Delsys	12 Delsys
Total Number of Movements (rest included)	53	50	50
Number of Movement Repetitions	10	6	6
			
*Exercise 1*			
Reference in Fig. 2	Exercise A	Exercise B	Exercise B
Number of Movements	12	17	17
Ground Truth Parameter	Hand Kinematics	Hand Kinematics	Hand Kinematics *(when available)*
Hand Kinematics/Dynamics Sensors	Cyberglove II	Cyberglove II	Cyberglove II *(when available)*
			
*Exercise 2*			
Reference in Fig. 2	Exercise B	Exercise C	Exercise C
Number of Movements	17	23	23
Ground Truth Parameter	Hand Kinematics	Hand Kinematics	Hand Kinematics *(when available)*
Hand Kinematics/Dynamics Sensors	Cyberglove II	Cyberglove II	Cyberglove II *(when available)*
			
*Exercise 3*			
Reference in Fig. 2	Exercise C	Exercise D	Exercise D
Number of Movements	23	9	9
Ground Truth Parameter	Hand Kinematics	Hand Dynamics	Hand Dynamics *(when available)*
Hand Kinematics/Dynamics Sensors	Cyberglove II	FFLS	FFLS *(when available)*

**Table 2:** Clinical characteristics of the amputated subjects.

**Subject**	**Handedness**	**Amputated Hand(s)**	**Amputation Cause**	**Remaining Forearm (%)**	**Years since Amputation**	**Phantom Limb Sensation (0–5)**	**DASH Score**	**Prosthesis Use**
1	Right	Right	Accident	50	13	2	1.67	myoelectric
2	Right	Left	Accident	70	6	5	15.18	cosmetic
3	Right	Right	Accident	30	5	2	22.50	myoelectric
4	Right	Right & Left	Accident	40	1	1	86.67	No
5	Left	Left	Accident	90	1	2	11.67	kinematic
6	Right	Left	Accident	40	13	4	37.50	kinematic
7	Right	Right	Accident	0	7	0	31.67	No
8	Right	Right	Accident	50	5	2	33.33	myoelectric
9	Right	Right	Accident	90	14	5	3.33	myoelectric
10	Right	Right	Accident	50	2	5	11.67	myoelectric
11	Right	Right	Cancer	90	5	4	12.50	myoelectric
